# Distinctive Processing Effects on Recovered Protein Isolates from Laurel (Bay) and Olive Leaves: A Comparative Study

**DOI:** 10.1021/acsomega.3c04482

**Published:** 2023-09-22

**Authors:** Hilal Yılmaz, Busra Gultekin Subasi

**Affiliations:** † Department of Biotechnology, Faculty of Science, Bartın University, 74100 Bartın, Türkiye; ‡ Faculty of Life Science, Division of Food and Nutrition Science, Chalmers University of Technology, 412 96 Gothenburg, Sweden

## Abstract

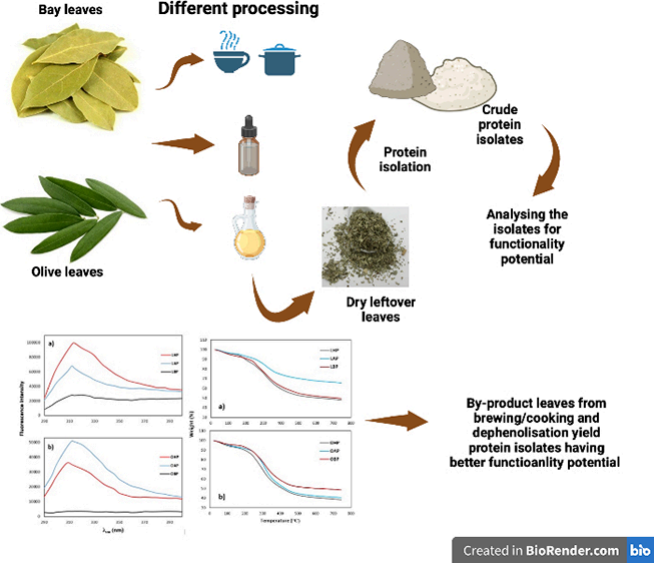

Although there is a well-known awareness of the nutritional potential of plant proteins, their utilization within food formulations is currently limited due to insufficient investigation of the functional properties or processing conditions. In this study, the protein contents of the remaining pulps of laurel (bay) (LL) and olive leaves (OL) after alcoholic washing (representing phenolic compound extraction), heat treatment (representing the usage of the leaves for tea brewing or as cooking aid), and deoiling process (representing oil extraction) were investigated. Bicinchoninic acid assay (BCA) indicated that the best protein yield was achieved with a direct isolation process after hexane oil removal. Both LL and OL isolates contained around 80% protein, but high temperature and alcohol content broke down the protein structure as well as decreased the final protein content (∼40%). Alcohol treatment appears to remove protein-bound phenols and increase fluorescence intensity in OL protein isolates while potentially causing structural alterations in LL proteins. In addition to a dramatic decrease in fluorescence intensity, the absolute zeta potentials of protein extracts of boiling OL and LL increased by 53 and 24%, respectively. The increased zeta potentials along with the decreased fluorescence intensity indicate the changes in the protein conformation and enhanced hydrophilicity of the protein structure, which can influence the functional properties of proteins. Protein extracts of deoiled LL had the highest Δ*H* value (180 mJ/mg), which is higher than other laurel and all olive protein samples. Laurel protein isolates became more thermally stable after hexane treatment. Moreover, the protein extracts after hexane treatment showed better emulsion capacity from both laurel (71.57%) and olive (61.87%). Water-binding capacity and thermal stability of the protein extracts from deoiled samples were higher than those of the other pretreatments, but the boiled samples showed higher oil-binding capacity due to protein denaturation. These findings indicate the importance of processing conditions in modulating protein properties for various applications.

## 1 Introduction

Sustainable and economical food production is one of the most important challenges to be solved for present and future generations. Great responsibility falls on industry and academia to manage and process food waste and byproducts. Biological conversion, extraction, and purification of desired compounds from foods’ byproducts are essential for “zero waste”.^1^ Evaluation of vegetable waste among food wastes has gained popularity. Following the industrial processing of distinctive plant-based raw materials for varying purposes, a concomitant production of waste/byproducts is inevitable. Among those crops, the production and processing of olive and laurel are being encouraged due to their economic potential which are yielding a vast amount of waste at the end of processing such as oil and phenolic compounds extraction.^2^

Proteins are valuable nutritional components with specific physicochemical and functional properties. Using plants as a protein source prevents the increase of greenhouse gas emissions, which is a natural but undesired outcome of animal-based protein sources such as meat, milk, and/or eggs.^3^ In addition, the required energy and water amount to be consumed for plant-based protein production are considerably lower than those of animal-based protein production.^4^ Despite their qualified protein contents in high quantities, animal-based protein sources are known to contain considerable amounts of deleterious components such as cholesterol and saturated fatty acids, which might cause cardiovascular diseases and even some cancer types if consumed frequently.^5^ With widespread awareness for better nutrition, plant proteins have risen in popularity among individuals recently. However, the use of plant-based proteins in food formulations is currently under desired levels. The reasons for this issue are considered as the digestibility/bioavailability and/or techno-functional properties of proteins obtained from plant-based sources have not been sufficiently decoded and/or improved. To utilize plant proteins as an ingredient for industrial food production, their functional properties such as foaming, emulsifying, and/or gel-forming properties should also be investigated.^6^

Laurel leaves (LL) (*Laurus nobilis* L.) contain 5–6% oil and 10–14% protein in their fresh forms.^7^ The oil extracts of laurel plants have significant economic value with an extensive potential to be used in varying application areas, particularly in the food, pharmaceutical, and cosmetic industries.^8^ On the other hand, LL itself has therapeutic effects and is used for its antibacterial, anti-inflammatory, antidiabetic, and antiseptic properties as well as against stomach ailments.^9−11^ Oils and other compounds such as anthocyanins that are obtained from laurel are used as natural dying pigments and flavoring agents.^12−16^ In recent years, LL have been used to produce some warm (as herbal tea) and cold-soft drinks worldwide in-house or industrially with varying recipes, particularly for their health and functionality-attributed properties.^17^ Following the brewing process, the remaining LL might still have an industrial potential in terms of protein content and require further investigation.

As another Mediterranean crop, olive is being processed into olive oil extraction by around 75% of its total annual production and yields an excessive amount of industrial waste that still contains distinctive valuable compounds. Byproducts of olive oil production consist of pomace, black water, olive leaves (OL), and branch fractions.^18^ Among these byproducts, OL constitute a substantial portion of the total harvested mass, accounting for approximately 5% of the olive fruits’ mass. As a result of the pruning of olive trees, approximately 25 kg of leaves and branches emerged from the tree. Even if, other olive fruit byproducts, such as pomace, are also valuable sources for waste management and bioactive compounds, these components are already widely extracted and utilized within the olive oil industry.^19,20^ By focusing on OL, we can diversify waste management strategies and ensure that all valuable components from the olive tree are utilized, reducing potential competition for the same resources. On the other hand, OL is known to contain bioactive compounds with various therapeutic properties, such as antibacterial, anti-inflammatory, and antidiabetic effects. Therefore, in recent years, consumption of OL has increased by processing in different ways such as boiling the leaves, brewing them as tea, or adding them to food as spice, or extracting their oil for cosmetic purposes like LL.^21,22^ After these leaves were processed and utilized, the remaining pulp and its protein content can be utilized to produce natural remedies and functional products.

Rather than considering LL and OL as mere waste, processing them allows for the recovery of valuable compounds. Among these, the most significant component is the protein content, which can be extracted and utilized for various purposes including food formulations, dietary supplements, and functional ingredients. The valorization of OL and the extraction of valuable compounds might open up new economic opportunities. The recovered proteins and other bioactive components have potential applications in the food, pharmaceutical, and cosmetic industries. Moreover, by transforming waste into valuable products, new revenue streams might be created and contribute to the country’s economy.

Following the application of different processing conditions, the protein content of LL and OL and the availability of these proteins have remained a question mark, especially with the increasing interest in plant-based protein sources in recent years. Since, protein content and techno-functional properties of obtained proteins recovered from LL and OL byproducts have not been adequately investigated, in this study, the protein contents of the remaining bio pulps following different processes such as alcoholic treatment, heat treatment, and deoiling were investigated representing phenolic compound extraction, heat treatment (leaves for tea preparation or cooking aid), and deoiling process (oil extraction), respectively. The protein content level and some physical and structural properties of the obtained protein isolates were examined with BCA assay, zeta potential measurement, and fluorescence spectroscopy. The functional properties, mainly the water and oil binding capacities, emulsifying and foaming properties of extracted proteins were analyzed. In addition, thermal characteristics using differential scanning calorimetry (DSC) and thermogravimetric analysis (TGA) revealed a piece of frontier information about the techno-functional potentials of proposed revalorized protein isolates. The thermal properties are crucial for understanding the behavior of proteins during processing and their potential applications in food and industrial settings.

## 2 Materials and Methods

### 2.1 Leaf Processing and Isolation of Proteins

LL and OL were collected from trees in the Bartın and Hatay regions in Türkiye, respectively. The leaves were dried in a conventional oven at 70 °C for 24 h and ground into fine powder. Then, the powders of raw dry LL and OL were sieved separately by 0.5 mm. Before protein isolation, three different processing conditions were applied to both LL and OL to mimic real-purpose domestic- and industrial-scale processing conditions.

#### 2.1.1 Brewing/Cooking Process (1)

Boiling water at 100 °C was added to the LL and OL samples and stirred for 5 min. Then grounded leaves were removed from the media, dried under the same conditions as fresh leaves, and stored before protein isolation at 4 °C for further use.

#### 2.1.2 Phenolic Compounds Extraction Process (2)

Samples were processed to mimic the phenolic compounds removal by adding 80% ethanol to grounded LL and OL samples and stirred for 5 min.^23^ Similar to the previous treatment, grounded leaves were removed from the media, then dried, and stored before protein isolation at 4 °C for further use.

#### 2.1.3 Oil Extraction Process (3)

For deoiling, approximately 300 mL of *n*-hexane was added to 5 g of each LL and OL samples connected to the extractor and condenser (Soxhlet extractor). The solvent flow rate was manually adjusted to 7 min/cycle during the extraction process, which was terminated after 4 h. Then, *n*-hexane was removed using a rotary evaporator under reduced pressure at 50 °C. The flasks containing the extracted oils were placed in a desiccator chamber for 1 h. The weights of the obtained oils were measured, and the yields were calculated.^24^ Following the completed procedure, deoiled leaves were removed from the media, then dried, and stored before protein isolation at 4 °C for further use.

After brewing/cooking, phenolic removal, and oil extraction processes, proteins were isolated using alkali extraction and acid precipitation technique.^2^ As starting material, 10 g of sample was mixed with 300 mL of 1% NaOH solution at room temperature for 1 h on a magnetic stirrer (300 rpm) and then centrifuged (2600 × *g* for 10 min at 4 °C). The collected supernatant was adjusted to pH 4.5 (isoelectric point) by adding 0.5 M HCl and mixed with a magnetic stirrer at 300 rpm for 30 min. At the end of this step, the precipitated proteins were collected by centrifugation (2600 × *g* for 10 min at 4 °C). The final protein isolates from the LL and OL were named laurel-boiling process (LBP) and olive-boiling process (OBP) for the boiling process, laurel–alcohol process (LAP) and olive–alcohol process (OAP) for the alcohol process, and laurel–hexane process (LHP) and olive–hexane process (OHP) for the hexane process, respectively.

### 2.2 Measurement of ζ-Potential

Protein solutions (0.5 mg/mL) were prepared with protein isolates of LL and OL after processing (1), (2), and (3) using distilled water. Zeta potential measurements of the protein isolate samples were determined by Zetasizer Nano ZS as a function of pH by the addition of 0.5 M HCl or NaOH as appropriate. (Malvern Instruments, Ltd., UK).

### 2.3 Bicinchoninic Acid (BCA) Assay and Absorption

In order to quantify proteins in a bulk solution, a BCA protein qualification assay was established. This technique is based on the reduction of Cu^2+^ to Cu^+^ in the presence of peptide bonds and subsequent complex formation with BCA to form a purple-colored end-product.^25^

### 2.4 Fluorescence Spectroscopy

All intrinsic fluorescence measurements were carried out using an FS5 Spectrofluorometer (Edinburgh Instruments, Livingston, UK) with a 150 W xenon lamp and a single photon counting photomultiplier (PMT) detector (Hamamatsu, R928P). The excitation wavelength range (λ_ex_) was at 280 nm, and the emission wavelength range was from 290 to 420 nm (measured every 2 nm). Other settings of the instrument were a slit width of 2 nm (for both excitation and emission) and a photomultiplier (PMT) detector voltage of 1245 V.

### 2.5 DSC and Thermal Analysis

TGA was carried out using a PerkinElmer Diamond TG/DTA Thermal Analysis instrument. The protein isolates of 5–10 mg were heated to 700 °C with a rate of 10 °C/min in a dynamic nitrogen atmosphere for TGA analysis. Thermal properties of DSC were analyzed using a Hitachi DSC 7020 (Minato-ku, Tokyo, Japan). Indium was used for instrument calibration, and dry nitrogen cell purge were applied with a 40 cc/min flow rate. Roundly 5 mg (dry basis) of samples was sealed in hermetic aluminum pans with an identical reference pan sample. The temperature range was screened between 20 and 300 °C with 10 °C/min steps. No sample loss was observed by following the procedure. Each sample was run in duplicate.

### 2.6 Functional Properties

#### 2.6.1 Water- and Oil-Binding Capacities

The water- and oil-binding capacities were determined using the standard method employed by Manamperi et al.^26^ The absorbed amounts of water and oil were determined by dividing the difference between the initial and final weights by the sample amount.

#### 2.6.2 Emulsion Capacity and Stability

The determination of emulsion capacity (EC) and stability (ES) was carried out based on the method established by Wu.^27^ Following a 7:100:100 (w:v:v) ratio, 1.05 g of defatted isolate was weighed and then 15 mL of distilled water was added. To the slurry, 15 mL of sunflower oil was added followed by mixing to determine EC using eq 1. For the assessment of ES, the samples were kept in an 80 °C water bath for 30 min. After the specified time, the samples were rapidly cooled under running water. The samples were then centrifuged for 5 min to determine the ES using eq 2.
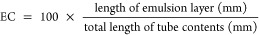
1
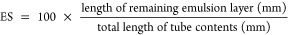
2

#### 2.6.3 Foaming Capacity and Foam Stability

Foaming capacity (FC) and foam stability (FS) were determined following the method that was established by Latif and Anwar.^28^ Sample dispersions were prepared using 3 g of defatted olive and laurel protein isolate in 100 mL of distilled water. The samples were shaken vigorously at high speed for 3 min at room temperature and quickly transferred to 250 mL graduated cylinders. The total volume and liquid volume were recorded immediately to determine the FC. After 30 min of standing at room temperature, the remaining foam volume was recorded to determine the FS. The FC and FS equations are presented with eqs 3 and 4, respectively.
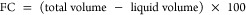
3
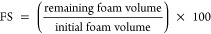
4

### 2.7 Statistical Analysis

Data obtained in this study were expressed as the mean ± standard deviation of triplicate measurements. Data were statistically analyzed for multiple comparisons using SPSS software (version 28, IBM SPSS Inc., Armonk, NY, USA) for analysis of variance (ANOVA). Duncan’s novel multiple-range test was applied to compare different samples, with significance established at *p* < 0.05.

## 3 Results and Discussion

### 3.1 Fluorescence Spectroscopy Investigations

**Figure 1:**
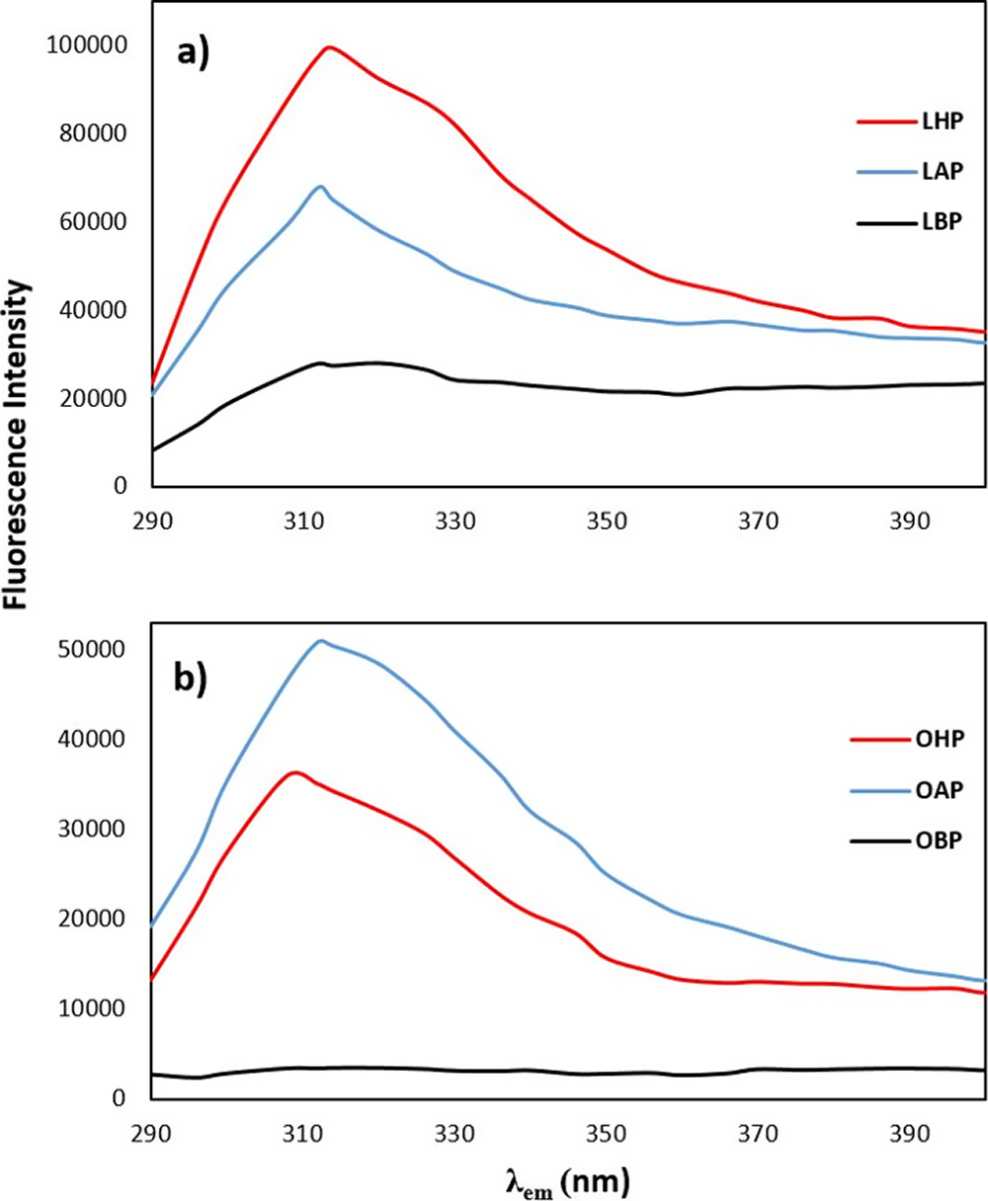
Fluorescence emission spectra (at λ_ex_ = 280 nm) of protein isolates from (a) laurel–hexane process (LHP), laurel–alcohol process (LAP), laurel-boiling process (LBP), and (b) olive–hexane process (OHP), olive–alcohol process (OAP), and olive-boiling process (OBP).

Fluorescence is the most popular technique that has been used to estimate conformational changes and binding properties of proteins. It depends on the intrinsic fluorophore of the tyrosine (Tyr), tryptophan (Trp), and phenylalanine (Phe) residues in the protein.^29^ However, the fluorescence emission of the proteins is dominated by Trp, which absorbs at the longest wavelength. In the presence of Trp, although there are Phe and Tyr amino acids in the protein, the energy that they absorb is mainly transferred to Trp. Protein fluorescence is generally excited at 280 nm, but Phe displays a structured emission with a maximum near 282 nm.^29^ Therefore, Phe, having a very small quantum yield, was not as excited as in this present study. The emission maximum of Tyr and Trp in water occurs at 303 and 350 nm, respectively. Thus, in Figure 1, the observed emission peaks were due to the absorption of both Tyr and Trp at 280 nm. On the other hand, resonance energy transfers repeatedly occur from Tyr to Trp, so only a minor contribution of Try to the emission of most proteins can be observed.

Figure 1 shows the effect of the processing conditions on the fluorescence emission spectra of the protein isolates from LL and OL. Broad and slightly shouldered peaks were observed for each protein isolate. This type of fluorescence intensity peak means the presence of a high amount of Tyr amino acids in addition to the Trp. On the other hand, LL protein isolates had higher fluorescence intensity (*F*_max_) than OL protein isolates at each processing condition (Table 1). Protein isolation after waiting for 5 min in boiling water dramatically decreased the fluorescence intensity of the protein isolated from both laurel (LBP) and olive leaves (OBP). While the fluorescence intensity of the protein isolates decreased after alcohol treatment in laurel leaves (LAP), an increase in the fluorescence intensity of OL protein isolates was observed after alcohol treatment (OAP). According to these results, it is estimated that treatment with alcohol increases the fluorescence intensity of OL proteins by removing protein-bound phenols. Treatment with alcohol probably induced the removal of some LL phenols, while decreasing the fluorescence intensity suggests that alcohol might cause alterations in the structure of LL proteins. The potential removal of phenolic compounds might decrease the number of protein–phenolic interactions in the structure and increase the formation of protein–protein interactions.^30^

**Table 1:** Zeta Potential, Fluorescence Intensity, and Total Protein Content of Protein Isolates from OL and LL^a^

sample	ζ-potential (mV)	λ_max_ (nm)	*F*_max_	BCA (mg/mL)
laurel–hexane process (LHP)	–28.4	314	99.33	0.82
laurel–alcohol process (LAP)	–32.2	312	67.83	0.38
laurel-boiling process (LBP)	–35.3	312	27.91	0.55
olive–hexane process (OHP)	–24.5	308	36.04	0.73
olive–alcohol process (OAP)	–28.7	312	50.74	0.44
olive-boiling process (OBP)	–37.5	314	3.47	0.44

a *F*_max_: maximum fluorescence intensity; λ_max_: maximum peak positions; BCA: bicinchoninic acid assay

In addition to the fluorescence intensity (*F*_max_), the maximum peak positions (λ_max_) were also changed with the changing processing conditions. For olive protein isolates, λ_max_ had a redshift up to 6 nm when exposed to alcohol (OAP) and boiling water (OBP) (Table 1). On the contrary, λ_max_ has a 2-nm blue shift for laurel after being processed with alcohol (LAP) and boiling water (LBP). As it is known and mentioned in the study (Lakowicz, 2006),^29^ the emission of indole can have a blueshift if the group is buried within a native protein (N). In the meantime, if an interaction or processing conditions cause protein unfolding, then a redshift is observed. The decrease in fluorescence intensity and redshift is attributed to the interaction and possible unfolding in fluorescence studies.^29^

It is known that heating proteins themselves can result in significant redshifts in fluorescence spectra. This is attributed to the unfolding of polypeptide chains, exposing hydrophobic residues, and making the proteins more accessible for ligand binding, thereby leading to decreased fluorescence intensity.^31^ In the present study, proteins were not heated directly. However, prior to protein isolation, the whole plant leaves were subjected to heating through boiling water, which still caused similar unfolding effects on the protein structure, as indicated by the fluorescence results.

### 3.2 Zeta Potential of the Protein Isolates

As shown in Table 1, the absolute values of the zeta potential for all samples increased after alcohol and boiling water treatment. Treating the OL with alcohol resulted in a 17% increase in zeta potential (more negative values) compared to the protein isolates obtained through the normal extraction process after oil removal with hexane. Boiling the OL increased the zeta potential of protein isolates by 53%. Similar behavior was observed for LL, with zeta potential values of LL protein isolates became more negative by 13 and 24% after alcohol and boiling treatment, respectively. The zeta potential values, along with the fluorescence results indicating a redshift in λ_max_, suggest that the increased presence of negatively charged residues may promote the unfolding of OL proteins. Similarly, LL protein isolates exhibited more negative zeta potentials after alcohol and boiling water treatment, coinciding with a decreased fluorescence intensity, which may imply changes in protein conformation. The increased absolute zeta potential and decreased fluorescence intensity also suggest enhanced hydrophilicity of the protein structure, potentially leading to improved protein solubility and enhanced techno-functional properties.^32^ Proteins are soluble when electrostatic repulsion is stronger than attractive forces (van der Waals or hydrophobic interactions). Conversely, protein insolubility near its isoelectric point (pI) is due to weak repulsive forces, promoting the growth of protein aggregates.^33,34^ It should be emphasized that a direct comparison and discussion of the present results with literature data are not possible, as there is no similar study available on the zeta potential of distinctively processed LL and OL protein isolates. However, it should be noted that zeta potentials have implications for the properties of the extracted proteins in terms of hydrophilicity, conformational changes, aggregation, and stability. The zeta potential is an indicator of the electrostatic repulsion between protein molecules. The higher absolute zeta potential values suggest the enhanced surface hydrophilicity and the stronger repulsive forces, which is important for preventing protein aggregation and precipitation, especially in food formulations.^35^

### 3.3 BCA Analysis of the Protein Isolates

The total extractable protein was 84 mg/g of dried OL material and 45 mg/g of dried LL material as a control sample without any preprocessing step. The highest protein yield was achieved through direct protein isolation after removing the oil with hexane (83.8 mg of protein/g of OHP and 44.2 mg of protein/g of LHP), which was followed by a boiling process (83 mg of protein/g of OBP and 44 mg of protein/g LBP). The lowest protein yield was obtained after alcohol pretreatment due to the possible loss of some proteins together with phenolics (33.8 mg of protein/g of OAP and 21.5 mg of protein/g LAP). Protein isolates from both LL and OL (LHP and OHP) contained approximately 80% protein. However, the protein content significantly decreased to around 40% purity in the isolates treated with alcohol and boiling water, as indicated by BCA analysis (Table 1). This decrease can be attributed to the elevated temperature and the presence of high alcoholic content, which disrupt the protein structure and consequently reduce the overall protein content. The results highlight the sensitivity of protein structure to processing conditions, particularly temperature and alcohol exposure. The decrease in protein content observed after alcohol and boiling water treatments indicates denaturation and degradation of proteins, leading to a lower final protein yield. These findings are consistent with previous studies that have shown how elevated temperatures can unfold protein structures.^36,37^

It is worth noting that the choice of processing conditions can significantly impact the protein quality and yield. While the direct protein isolation process after oil removal with hexane resulted in the highest protein content, alternative treatments involving alcohol and boiling water led to a substantial decrease in protein purity. This emphasizes the importance of optimizing processing parameters to achieve the desired protein characteristics for specific applications.

Utilizing the BCA assay for protein content assessment in crude protein mixtures is a common approach, yet it is crucial to consider potential interferences that could impact result accuracy and reliability, particularly in complex mixtures containing various components. In complex protein mixtures, the presence of nonprotein components like lipids and carbohydrates can affect BCA assay accuracy, potentially leading to protein content overestimation or underestimation. As the BCA assay relies on the availability of the protein’s peptide bonds for the reduction of Cu^2+^ ions, denatured or partially unfolded proteins may expose more peptide bonds, leading to increased color formation and potentially overestimating the protein content.^38^

Despite these potential interferences, the qualitative comparison of the protein content in this study is reasonable and informative. This study primarily aims to compare the protein content after different processing treatments, and the observed trends of decreasing protein purity after alcohol and boiling water treatments are consistent with the expected denaturation and degradation of proteins under elevated temperature and alcohol exposure. Therefore, to compare protein content qualitatively, these potential interferences may not have a significant impact on the overall findings.

### 3.4 Thermal properties by DSC

DSC and thermal analysis can be effectively linked to the functional properties of proteins extracted from plant sources. These techniques provide valuable information about the thermal behavior of proteins, which is crucial for understanding their functionality and potential applications.

The thermal properties of OL and LL protein isolates were determined using DSC as shown in Table 2. DSC thermograms of LL and OL protein isolates after hexane, alcohol, and boiling treatments were also provided as Supporting Information (Figures S1 and S2). The curves in the temperature range of 40–80 °C indicate denaturation of the protein structures.^39^ Thus, it could be used to evaluate the thermal stability.^40^ The DSC results reveal the denaturation temperatures (*T*_peak_) and enthalpies (Δ*H*) of the protein isolates. Higher denaturation temperatures and enthalpies indicate better thermal stability, suggesting that the proteins can withstand heat treatment during processing without significant structural changes.^41^ This thermal stability is essential for various food applications as it ensures that the proteins retain their functionality during cooking, baking, or other thermal processing.

**Table 2:** Thermal Properties of Protein Isolates from OL and LL^a^

	**DSC**		**TGA**		
	*T*_peak_ (°C)	Δ*H* (mJ/mg)	first stage degradation (%)	second stage degradation (%)	*T*_max_ (°C)
**LHP**	74.7	180	4.07	47.50	316.79
**LAP**	75.9	106	4.46	29.76	311.13
**LBP**	71.5	92.2	6.04	43.94	314.43
**OHP**	79.9	51.7	6.23	54.72	298.28
**OAP**	70.0	57.5	6.23	52.70	310.99
**OBP**	73.7	54.9	4.20	46.52	313.66

a DSC: differential scanning calorimetry, TGA: thermogravimetric analysis, Δ*H*: denaturation enthalpy.

All samples showed an endothermic peak in the range 70–80 °C. Samples from LL had quite close denaturation temperatures, while only LBP had a slightly lower *T*_peak_ value (71.5 °C) compared to that of the other two treatments. However, the denaturation enthalpies of all laurel samples were different than each other and LHP had the highest Δ*H* value (180 mJ/mg). Laurel protein isolates became more thermally stable after hexane treatment (LHP). On the other hand, the differentiation for both denaturation temperature and enthalpy of OL-derived products was smaller. Among the OL samples, OHP and OAP seemed to have the highest *T*_peak_ and Δ*H* values, respectively, while OBP exhibited a so-called “average” value for both *T*_peak_ and Δ*H* of the other two samples. For LL samples, LHP might be proposed as the most thermal stable sample, but a similar deduction could not be made for OL samples; however, small data variation might be pointed out that OHP and/or OBP could induce the most thermally stable structures. More accurate comparisons and judgments could be possible when these data were accompanied by another complementary analysis like TGA. A similar case was observed by Feyzi et al.^42^ in their study covering the thermal stability comparisons of fenugreek protein isolates obtained from defatted raw materials using different solvents. They indicated that hexane-defatted fenugreek protein isolate had the highest *T*_peak_ and the lowest Δ*H* values and further considered that the Δ*H* value could be a more determinative parameter for thermal stability. It was also stated in another study that degradation onset temperature and Δ*H* values are more straightforward data for thermal stability comparison.^43^

### 3.5 Thermal properties by TGA

Since the thermal characteristics of a protein are closely related to its use in food industrial applications, TGA is widely used to understand protein stability and thermodynamic performance. The increase in the temperature causes a variety of changes to the protein samples such as the loss of free and crystalline water, evaporation of water, unleashing of small molecular volatiles, or oxidative breakdown of protein.^44^

**Figure 2:**
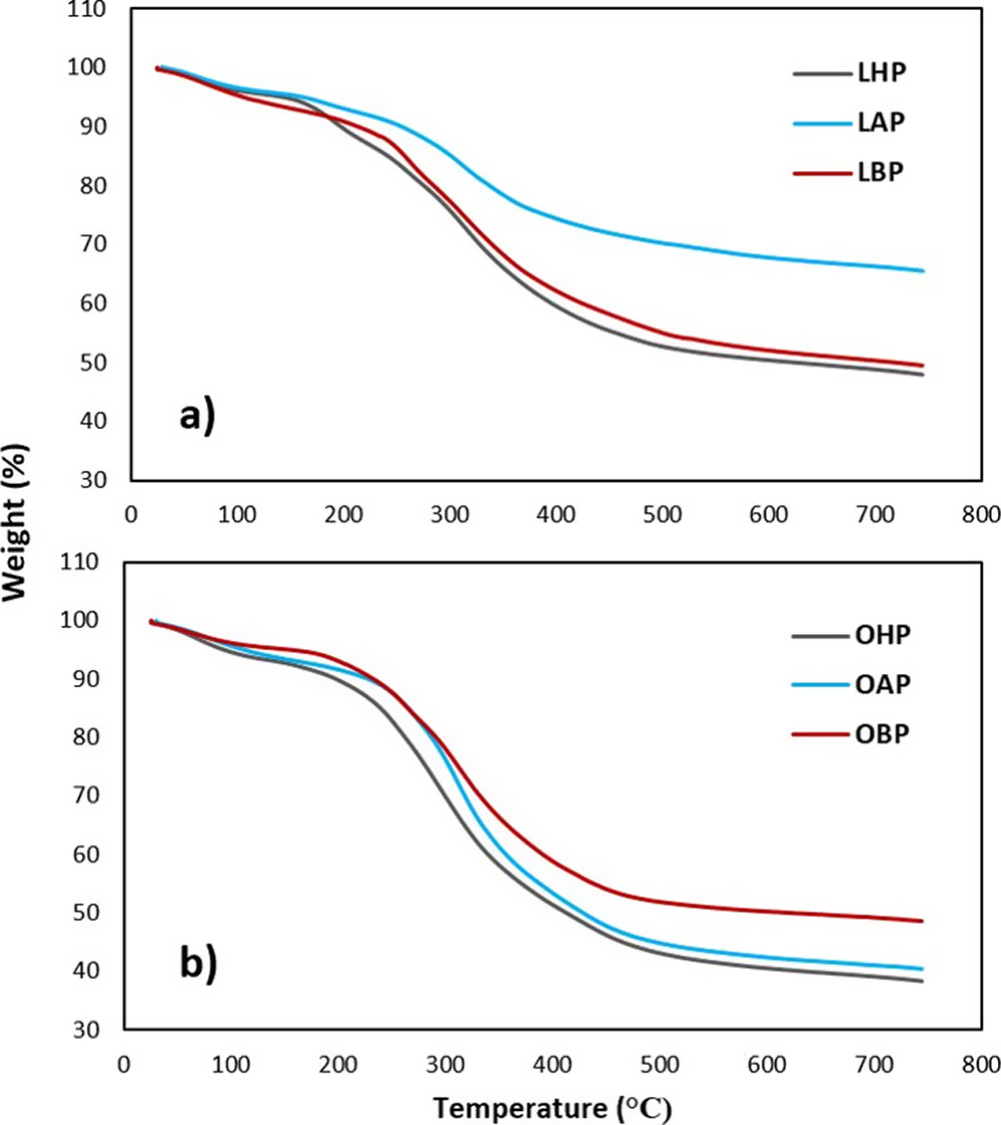
Thermogravimetric analysis (TGA) curves (weight loss) of the protein isolates from laurel (a) and olive (b) leaves.

**Figure 3:**
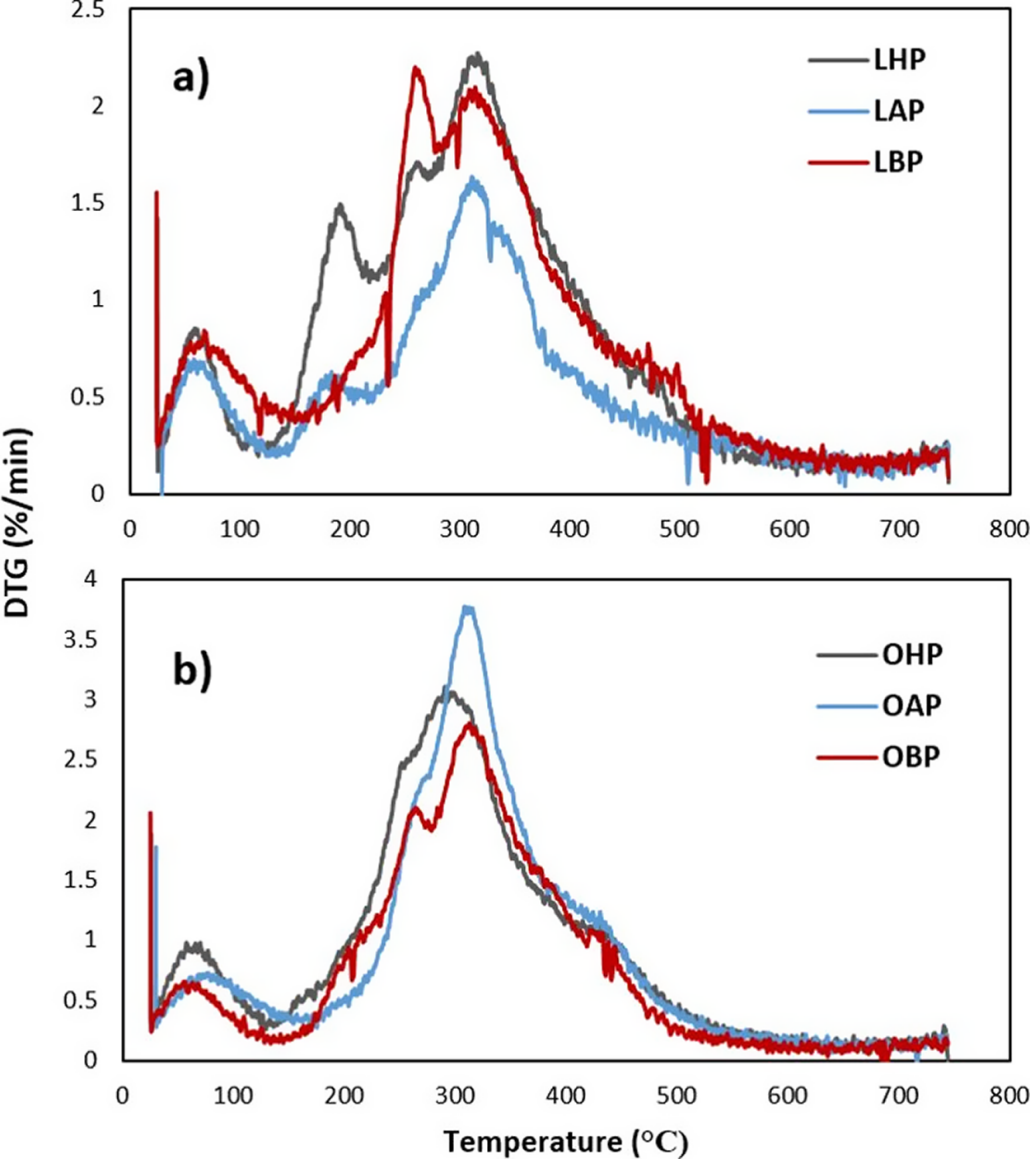
Derived thermogravimetric (DTG) curves (derived weight loss) of the protein isolates from laurel (a) and olive (b) leaves

The TGA curves for protein isolates from LL and OL after hexane, alcohol, and boiling treatments about the mass loss are shown in Figure 2 and their derived thermogravimetric (DTG) variation curves are shown in Figure 3. All samples showed similar thermograms where the mass loss processes were divided into two main stages during the scanning between 25 and 750 °C. The first stage in the temperature below 200 °C was associated with the loss of free and bound water from the protein molecules, which is due to the evaporation of free water and some other volatile compounds. The solubility of proteins is influenced by the presence of water and other volatile compounds, and these results can provide insights into the protein’s hydration properties. In the second stage (200–500 °C), all samples degraded rapidly with a large mass loss during thermal decomposition, which was related to subsequent component volatilization of proteins at the melting point due to the disruption of interactions (intra- and intermolecular hydrogen bonds, van der Waals forces, and electrostatic interactions). This type of temperature-dependent weight loss profile of the protein samples that were observed in this study was compatible with the literature.^45^ When the different processing conditions were compared, it was observed that the boiling resulted in a significant loss of the thermal stability of the protein isolates followed by alcoholic treatment. Considering the TGA results of DSC analysis, pretreatments with boiling and alcohol yielded the most thermally unstable protein structures for OL and LL isolates, respectively. It should be indicated that hexane treatment of LL and alcohol treatment of OL could be proposed as secondary significant processes, and in-depth investigations could be helpful to finalize this phenomenon. As a common process, alcohol pretreatment has a significant potential to observe less thermostable protein isolates with better techno-functional properties. Alcohol treatment was also suggested in another study as a useful process to obtain defatted and less heat-resistant protein isolates from rice bran.^46^

Moreover, thermal analysis can also provide information about the water-binding capacity (WBC), emulsification, and foaming abilities of proteins. Therefore, these results can be used to study the phase transitions that affect functional properties. For example, denaturation of proteins can promote or hinder the formation and stability of emulsions and foams, and water retention which is essential for texture formation and juiciness.

### 3.6 Water and Oil-Binding Capacity

The ability to retain water, known as water-binding capacity (WBC), can be influenced by factors such as the types of amino acids present, the shape of the protein molecules, and the balance between surface polarity and hydrophobicity.^47^ WBC plays a crucial role in foods with a thick consistency, such as soups and confectionery items, as well as in baked goods such as bread and cakes. In these products, water must be absorbed without causing the proteins to dissolve, ensuring the desired thickness or viscosity.

The WBC of protein extracts under different pretreatments is displayed in Table 3. The WBC of LL protein extracts was higher than the OL protein extracts. The highest WBC was obtained for LHP (3.65 g/g) and the lowest one was for OHP (2.67 g/g). When the WBC results were considered together with results from thermal analysis, it was observed that proteins with higher thermal stability tend to have better WBC. This means that the structural integrity of the proteins of LL might be maintained at higher temperatures, allowing them to retain their ability to interact with and bind water molecules efficiently.

**Table 3:** Functional Properties of Protein Isolates from LL and OL^a^

	LHP	LAP	LBP	OHP	OAP	OBP
**WBC** (g/g)	3.65 ± 0.23	3.15 ± 0.17	2.96 ± 0.21	2.67 ± 0.16	2.76 ± 0.18	2.70 ± 0.15
**OBC** (g/g)	2.18 ± 0.12	2.24 ± 0.11	2.35 ± 0.09	1.75 ± 0.08	1.20 ± 0.10	1.82 ± 0.08
**EC (%)**	71.57 ± 1.70	56.48 ± 1.20	45.78 ± 1.10	61.87 ± 1.50	38.46 ± 1.30	46.15 ± 1.20
**ES (%)**	34.25 ± 0.90	22.12 ± 1.10	19.56 ± 0.90	32.25 ± 1.60	15.38 ± 1.10	19.23 ± 0.90
**FC (%)**	50.00 ± 1.80	42.75 ± 1.40	41.27 ± 1.40	36.00 ± 1.20	31.90 ± 1.30	37.39 ± 1.20
**FS (%)**	3.6 ± 0.170	3.5 ± 0.180	2.8 ± 0.17	4.3 ± 0.09	3.8 ± 0.12	3.1 ± 0.09

a WBC: water-binding capacity; OBC: oil-binding capacity; EC: emulsion capacity; ES: emulsion stability; FC: foaming capacity; FS: foaming stability.

Oil-binding capacity (OBC) is the binding of oil by nonpolar side chains of proteins, which can also reflect the hydrophobic capacity of protein.^48^ The OBC of LL protein extracts was slightly higher than that of OL (Table 3). For each plant sample, the boiling process showed a slightly higher OBC, which could be due to protein denaturation. Denatured proteins may have altered surface properties and more accessible hydrophobic interactions, leading to increased oil binding capacity. Fluorescence and thermal analysis results also showed protein denaturation, and especially fluorescence spectrocsopy results indicated the changing solvent exposure of hydrophobic aromatic residues of the proteins such as Tyr and Trp. When proteins denature, their three-dimensional structure can unfold or change, potentially exposing hydrophobic regions that would otherwise be buried within the protein’s native structure. These exposed hydrophobic regions can bind more willingly to oil molecules, leading to increased OBC.^32^

### 3.7 Emulsion and Foaming Properties

Functioning as surfactants, proteins reduce surface tension and establish a viscoelastic zone at the interface between air and water, which is an important parameter in terms of the emulsion and FC of the proteins.

The emulsifying characteristics of a protein are evaluated through two important factors: the EC and the ES. EC quantifies a protein’s capability to produce an emulsion, while ES measures its capacity to maintain a stable emulsion over a specific period.^49^ The protein extracts after hexane pretreatment showed better EC for both laurel (71.57%) and olive (61.87%) leaf proteins (Table 3). However, for all the samples that were incubated in a hot water bath for 30 min and rapidly cooled, the amount of emulsion decreased by half. These results together with the decreased thermal properties and vanished fluorescence intensity, therefore, may be attributed to the denaturation of the proteins rather than just unfolding due to the boiling process. Highly denatured proteins are more prone to aggregate and aggregated proteins may form larger complexes that are less effective at stabilizing emulsions, as they may not be able to evenly coat the droplets.^50^

Meanwhile, when the FC and FS were evaluated, the protein extracts from LL had better FC than OL, but for all the samples FS was significantly low. The lower FC of the protein samples indicates that these proteins are less effective at trapping and stabilizing air bubbles within a liquid, which is essential for creating and maintaining foams in various applications. This reduced ability to form and maintain a foam can be attributed to factors such as protein denaturation, altered surface properties, or disruptions in the protein’s structure.^50^

## 4 Conclusions

Before using plant proteins in areas such as food, medicine, and cosmetics, knowing their stability, folding, and interaction properties under different processing conditions and especially in temperature changes facilitates the more effective (target-oriented) use of these proteins. From this point of view, in this study, protein isolates from LL and OL were exposed to different processing conditions and characterized by thermal and spectroscopic methods.

It was observed that different processes had different effects on laurel and olive leaf proteins. While the proteins obtained after hexane extraction of laurel showed a better thermally stable behavior, the same impact for OL was observed as a result of alcohol treatment. Thermal, fluorescence, zeta potential, and BCA results revealed that boiling and alcoholic treatments led to protein unfolding in both leaf samples. Alcohol treatment enhanced protein–protein interactions in olive samples, resulting in protected fluorophores and higher fluorescence intensity. Hexane-treated samples showed a better functionality. Boiling caused protein denaturation, leading to reduced thermal properties and fluorescence intensity, rendering the protein nonfunctional. The results underscore the potential of deoiled plant byproducts and their protein extracts as promising functional ingredients for various industrial applications. Furthermore, these findings emphasize the pivotal role of processing conditions in tailoring protein properties to suit diverse applications.

It is certain that more accurate quantification of individual protein content can be obtained by additional techniques such as SDS-PAGE, HPLC, or mass spectrometry. Nonetheless, for the scope of the study in question, the qualitative comparison achieved through the BCA assay, fluorescence, and thermal analysis appears to be sufficient to draw relevant conclusions about the impact of different processing conditions on the protein content.

Owning medicinal and aromatic properties, in-house consumption by individuals for centuries, and their future utilization potential nominate OL and LL byproducts as promising protein sources, particularly for Mediterranean countries. Since there is a huge lack of information about the techno-functional properties of OL and LL protein isolates, more studies are required for a comprehensive mapping of their utilization potential and economic feasibility. Further studies could explore alternative processing methods or modifications to mitigate the negative effects of high temperature and alcohol exposure on protein integrity. Additionally, evaluating the functional properties of the protein isolates under different processing conditions would provide valuable insights into their potential applications in various industries such as food and pharmaceuticals.

## Supporting Information Available

Differential scanning calorimetry thermogram of laurel leaves protein isolates after (a) hexane (LHP), (b) alcohol (LAP), and (c) boiling processes (LBP) (Figure S1); differential scanning calorimetry thermogram of olive leaves protein isolates after (a) hexane (OHP), (b) alcohol (OAP), and (c) boiling processes (OBP) (Figure S2) (PDF)
