# Supplementary material for: Distinctive Processing Effects on Recovered Protein Isolates from Laurel (Bay) and Olive Leaves: A Comparative Study
Source: ACS Omega. 2023 Sep 22;8(39):36179–87. doi: 10.1021/acsomega.3c04482 (PMC10552139; doi:10.1021/acsomega.3c04482)
Supplement: Supplementary file 1 — ao3c04482_si_001.pdf [file ao3c04482_si_001.pdf]

# Distinctive processing effects on recovered protein isolates from laurel (bay) and olive leaves: A comparative study

Hilal Yilmaz [1], Busra Gultekin Subasi [2,*]

[1] Department of Biotechnology, Faculty of Science, Bartın University, 74100 Bartın, Türkiye

[2] Faculty of Life Science, Division of Food and Nutrition Science, Chalmers University of Technology, SE-412 96 Gothenburg, Sweden

*Correspondence: subasi@chalmers.se

**Supplementary Figures**

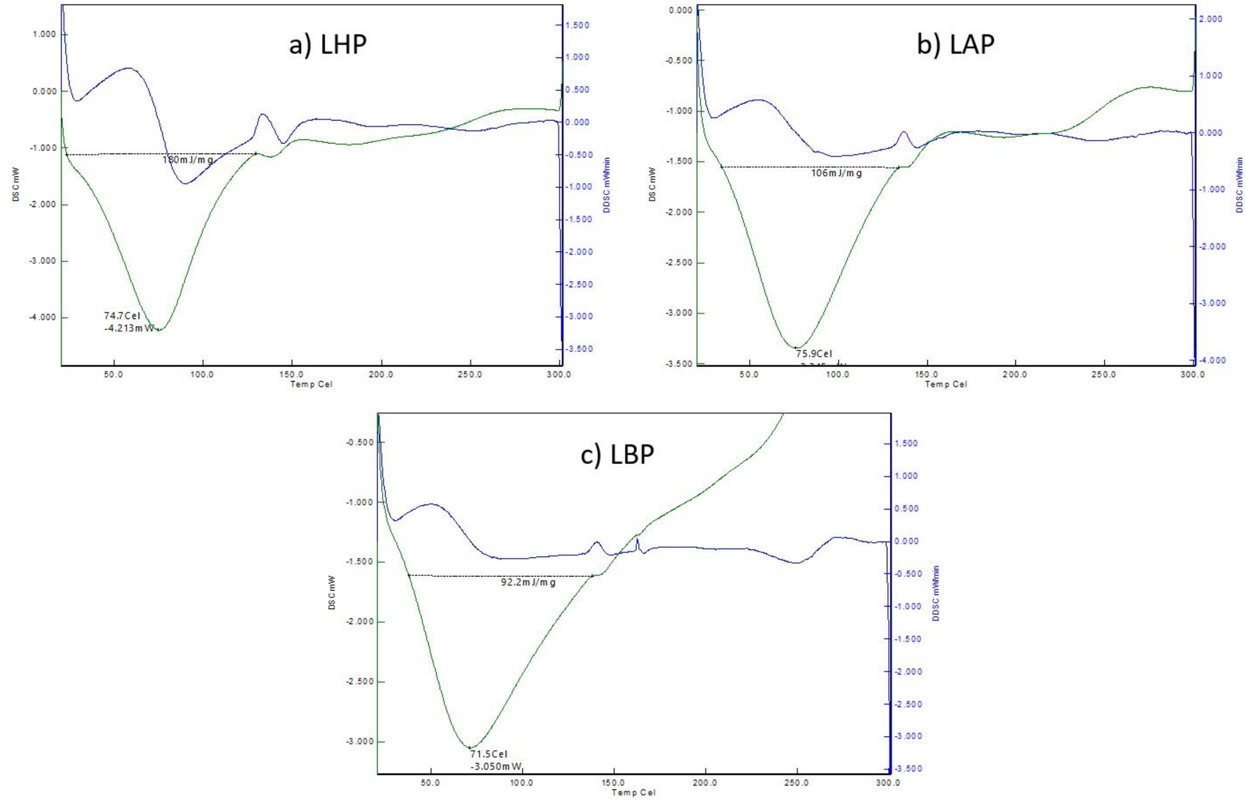


**Figure S1.** Differential Scanning Calorimetry thermogram of laurel leaves protein isolates after (a) hexane (LHP), (b) alcohol (LAP), and (c) boiling processes (LBP)

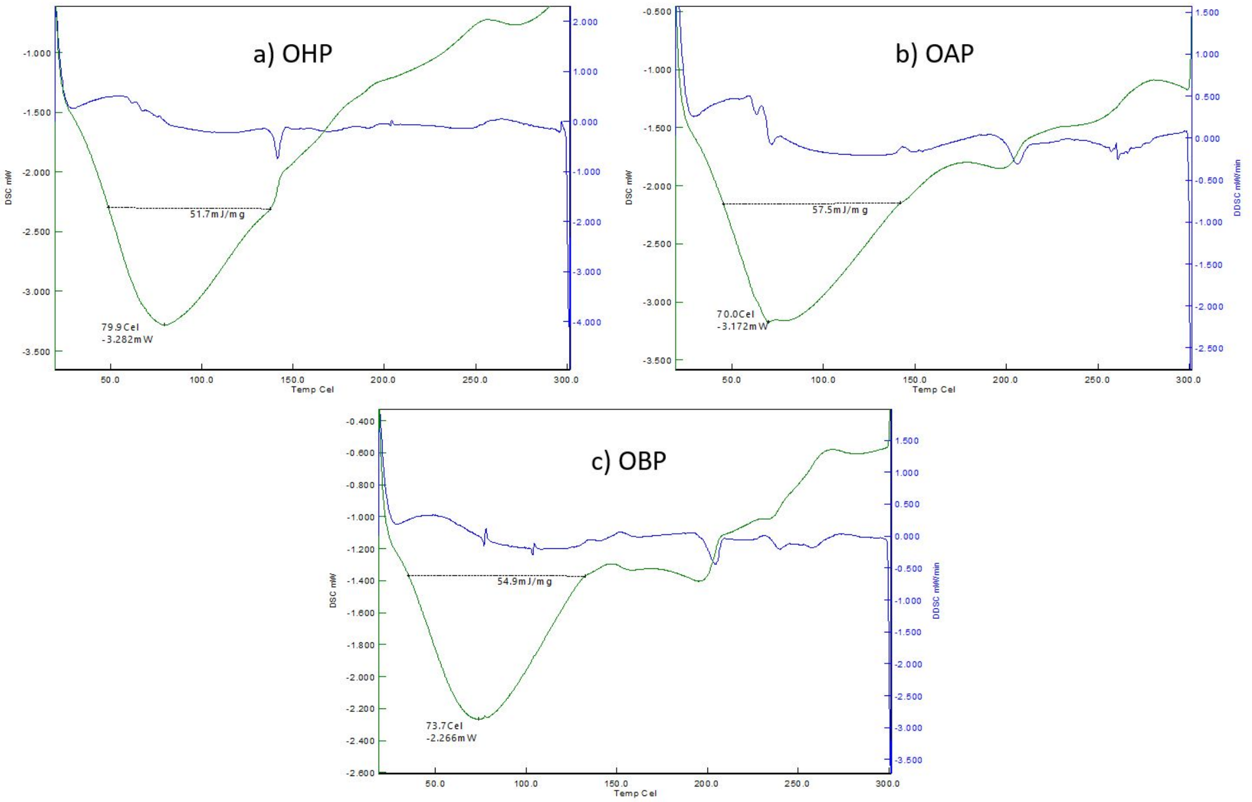


**Figure S2.** Differential Scanning Calorimetry thermogram of olive leaves protein isolates after (a) hexane (OHP), (b) alcohol (OAP), and (c) boiling processes (OBP)
